# Peptide Nucleic Acids Promise New Therapeutics and Gene Editing Tools

**DOI:** 10.1021/acscentsci.3c00016

**Published:** 2023-01-17

**Authors:** Rachel Brazil

DNA is the poster child for high-specificity binding. As long as their base sequences match, two complementary strands of DNA can navigate through a sea of biomolecules, find each other, and hold fast for millennia.

Three decades ago, chemists created a synthetic family of DNA-like molecules, peptide nucleic acids (PNAs), that bind even more strongly to nucleic acids and are not broken down in the body by enzymes that target DNA or RNA. These qualities could make them potent therapeutics aimed at silencing or editing genes.

PNAs are long molecular strands of nucleobases, much like DNA. But unlike DNA, their backbones are made from amino acids rather than sugar phosphates. “It’s combining two distinct fields, peptides and nucleic acids,” says Dan Appella, a chemist at the National Institute of Diabetes and Digestive and Kidney Diseases.

Today, one PNA drug is in clinical trials, and others are in development. But the path to making viable PNA therapeutics has been tough. Scientists in academia and start-ups have had to find chemical tweaks to PNAs’ backbones that allow the molecules to sneak into cells more easily and latch on to RNA more strongly to silence genes. Parallel to the development of these therapeutics, chemists are now able to harness PNAs’ ability to unzip and slip inside a DNA helix, making them ideal tools for gene editing and a potential alternative to the groundbreaking CRISPR-Cas9 system. Even though CRISPR has a decade’s head start, several gene-editing methods using PNAs are now in the works.

**Figure:**
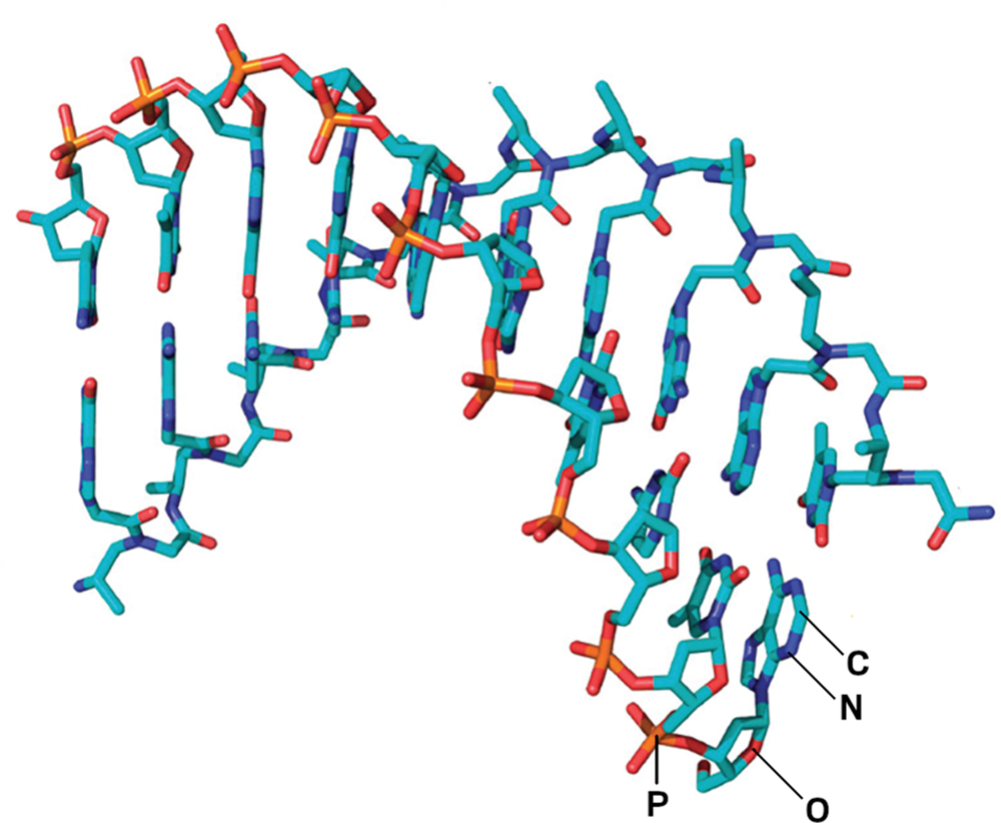
A peptide nucleic acid (turquoise backbone) can bind to a strand of DNA (orange backbone) if their nucleobase sequences are complementary. Credit: *J. Am. Chem. Soc*.

Many researchers are convinced that PNAs can provide superior therapeutics and a new editing tool, although their delivery to cells and to the right tissue in the body still needs to be improved. “If we can get these big molecules to be delivered appropriately and explore more of the biology, there is a big promise,” says Anisha Gupta, a PNA researcher at the University of Saint Joseph in Connecticut.

## How PNAs Work

The first PNAs were designed in 1991 by University of Copenhagen chemist Peter Nielson as his team was looking for molecules that mimic the way proteins bind to DNA. In addition to the Watson–Crick base pairing usually seen between complementary nucleic acid strands, Nielsen found that PNAs could bind to the outside of double-stranded RNA or DNA by forming hydrogen bonds to bases through a double helix’s major groove. This so-called Hoogsteen pairing created a triple helix.

But PNAs showed an even more fascinating binding power. “The real surprise was when we started doing binding assays and studies on double-stranded DNA. Instead of binding in the major groove, the first [PNA] molecules invaded the helix,” Nielson says. The PNAs were able to pull apart the tight embrace between DNA strands and slip inside. The lack of repulsion from PNAs’ neutral peptide backbone—compared with the repulsion caused by DNA’s negatively charged phosphate backbone—gave the PNA an edge in binding; in some cases, a PNA fully replaced one of the complementary DNA strands.

And PNAs have another superpower. They are not degraded by nuclease and protease enzymes the way synthetic DNA or RNA are, a property that grants them great long-term stability, says Appella, who has been designing PNAs for 20 years. Plus, PNAs can be made easily using the same solid-phase synthesis methods used to make other peptides.

## Boosting Performance

These qualities gave PNAs the ideal profile to act as antisense drugs, which bind mRNAs to smother protein production. Theoretically, PNAs can bind more strongly than most antisense drugs being developed today, which are snippets of DNA or RNA. But realizing that application was far from straightforward. “The hope was that the original PNAs would take us all the way, but I think it’s fair to say that some more chemistry needed to be done,” Appella says.

The first generation of PNAs was not soluble in water, so the molecules had an unfortunate tendency to clump up in solution and bind nonspecifically with other biopolymers in cells, leading to toxicity.

Danith Ly of Carnegie Mellon University and his colleagues addressed this limitation in 2006 when they refashioned PNAs’ peptide backbones to improve their ability to bind selectively to nucleic acids. By simply adding a substituent like a hydroxymethyl group a few carbons down the peptide chain from the nucleobase—at the γ position—they changed PNAs’ shape from a randomly folded molecule to an ordered helix stabilized by base stacking. The modified PNAs fit together with helical DNA and RNA like a glove, binding only to exact matches of their nucleobase sequence. Complexes of DNA and these so-called γ-PNAs showed a massive boost in stability compared with unmodified PNA complexes.

**Figure:**
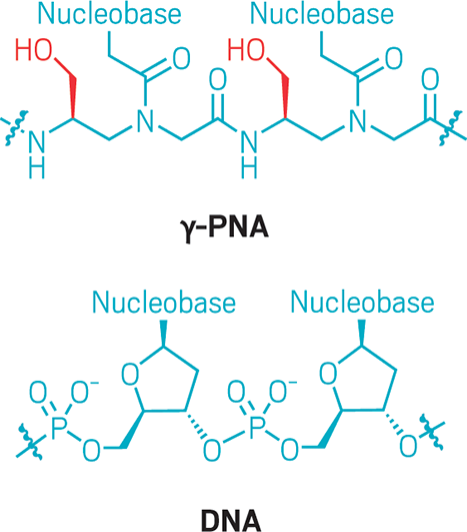
By adding a substituent such as a hydroxymethyl group at the γ position on a PNA backbone (red), Danith Ly’s group found they could increase the PNAs’ stability.

Since then, others have made their own souped-up PNAs with various goals in mind. Gupta addressed the question of solubility during her PhD research in Ly’s lab. By tuning the number of diethylene glycol side chains on the peptide backbone, she increased PNAs’ binding strength and tailored the solubility, which helped solve issues in formulating the PNAs and getting them to their target in the body, says Gupta.

Another challenge was transporting the molecules into cells. One way of doing this is attaching cell-penetrating peptide molecules to the PNA to shuttle them into cells, a method previously used to deliver nucleic acid drugs. Gupta approached the problem a different way: by packaging PNAs into polymer nanoparticles, which can slip into cells via natural processes, in which particles are surrounded and internalized by a part of the cell membrane. She treated tumors in mice with nanoparticles containing PNAs targeted to a cancer-associated RNA molecule. Although the cancer was not completely stopped, tumor growth slowed to about one-fifth of that in untreated mice.

Appella has been exploring another clever modification: adding tetrahydrofuran rings into the backbones of his PNAs. The new molecules enter cells much more easily, though he is not sure exactly why. His team used the approach to target RNA molecules known to be overexpressed in some tumors in vivo.

Ly, too, has continued to make innovations in PNA chemistry. He has moved on from altering the backbone to modifying the chemical structure of the nucleobases attached to it. He has designed two-ended nucleotide-mimicking molecules that can bind to both sides of a DNA or RNA helix, allowing for even stronger PNA binding and better gene silencing. He calls the double-ended molecules “Janus bases,” and he’s betting on them to push PNAs into new applications.

Ly envisions being able to use these to design PNAs that not only target base sequences but recognize and bind to secondary structures like RNA hairpins, opening up new possibilities in drug design.

In 2018, Ly showed that a Janus PNA can disrupt the mutant RNA hairpin that causes the neurological condition Huntington’s disease. By using Janus PNAs to bind to the hairpin’s characteristic repeating three-base sequence, Ly’s group hopes to prevent the hairpin from binding to and trapping proteins, an interaction that is thought to cause the disease’s symptoms.

“I think the holy grail of PNA application is probably going to be removing the natural nuclear bases and appending them with these Janus nuclear bases that we’re working on right now,” Ly says.

## PNA Drug Prospects

For now, though, the lead candidates for PNA-based drugs use a straightforward antisense approach. The South Korean drug company OliPass is currently the furthest ahead, with its most advanced drug candidate, the painkiller OLP-1002, now in clinical trials.

Olipass’s PNAs are modified with bases containing cationic lipid groups that increase stability and ease entry to cells. That makes them active in animal models at doses as low as 10 ng/kg, many orders of magnitude lower than existing antisense oligonucleotides.

The PNAs can pass into the nucleus and interact with pre-mRNA, the first version of mRNA produced by transcription before splicing enzymes excise the needless parts, says the company’s founder, Shin Chung. By binding to these pre-mRNA molecules, the PNAs essentially prevent splicing enzymes from cleaning up the RNA code, leading to mRNA that can’t be properly translated and thus preventing protein production.

OLP-1002 injections are currently in an Australian phase 2a clinical trial for treating osteoarthritis. The drug inhibits expression of the sodium ion channel, Na_v_1.7, known to be involved in pain. Unlike anesthetics such as lidocaine that affect various sodium ion channels, the sequence-specific PNA is selective and doesn’t risk shutting down sodium channels that control heart function, theoretically making it much safer.

The second company exploiting PNA technology is Pittsburgh-based Neubase Therapeutics, cofounded in 2009 by Ly with biotech entrepreneur Stephan Dietrich. The company now has PNA drug candidates in three diseases. Their most advanced candidate, NT-0200, treats myotonic dystrophy type 1, a progressive muscle disorder caused by faulty RNA that traps critical splicing proteins and leads to mistranslation in cells.

NT-0200 restores downstream protein production across a broad range of tissues, according to Neubase’s mouse studies. The company also has a PNA drug candidate that targets the Huntington gene’s repeating three-nucleotide sequence after it’s transcribed into mRNA.

## Gene Editing: The New Opportunity

But therapeutics targeting RNA may not be the first place PNAs will deliver. In October 2022, Neubase announced it would pivot away from its antisense drug program and instead focus its PNA operation on gene editing. In a partnership with an undisclosed global healthcare company, Neubase plans to create PNAs designed to edit genetic mutations in three undisclosed diseases. Biotech analyst Hartaj Singh from investment bank Oppenheimer describes the move as “a tactical retreat” from their antisense program to the tantalizing high-reward prospect of gene editing.

In academia, Ly—who no longer works with Neubase—along with Mark Saltzman and Peter Glaser of Yale School of Medicine are also collaborating on gene editing using PNAs. The method takes advantage of PNAs’ ability to invade and pry open a double-stranded DNA molecule, discovered by Nielson back in 1991. In the new approach, two PNAs connected with a linker molecule can huddle around a DNA helix and create a small opening in the helix about five or six bases long. “Then the PNA snakes in to bind to one of the strands [of DNA], and the other strand will be looped out,” Ly explains. The bases on that liberated DNA strand are then exposed and accessible for editing.

**Figure:**
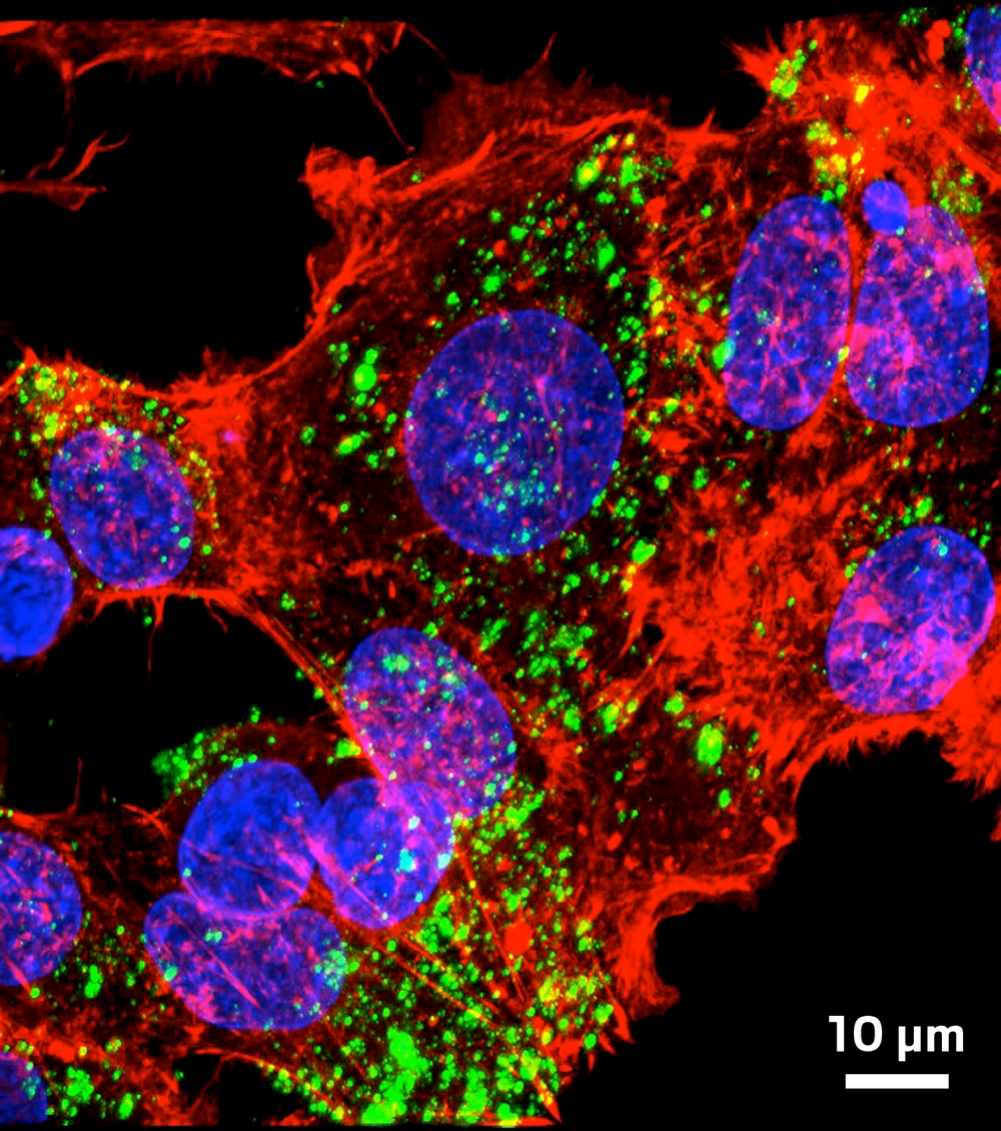
Danith Ly, Mark Saltzman, and Peter Glaser's polymeric nanoparticles (green flecks) enter cells loaded with γ-PNA machinery that can edit genes. Credit: Christopher Cheng.

Unlike with CRISPR, there is no need for sneaking an accompanying enzyme like Cas9 into the cell; the structure that the PNAs form recruits the cell’s own DNA repair machinery. So when the researchers provide the desired DNA insertion, recombination takes place naturally, correcting the gene, Ly says. As for a delivery method, Saltzman’s team has created biodegradable nanoparticles containing PNA and guide DNA that correct mutations causing cystic fibrosis in mouse models—both when inhaled and when injected intravenously.

Chemist Yi Lu of the University of Texas at Austin developed a similar gene-editing method called PNA-assisted double-stranded DNA nicking by DNAzymes (PANDA). He is also using a PNA to open up double-stranded DNA, but he uses a DNAzyme—a DNA molecule capable of cleaving a single DNA strand—to elicit the natural repair process. Lu has shown his system can provide accurate editing by targeting differences as small as one base.

Currently, though, gene editing using CRISPR is way ahead of these PNA methods. “Our editing efficiency is low,” Saltzman admits. Their work correcting the cystic fibrosis gene has reached 9% efficiency when inhaled and only 1% when injected in mice. Efficiencies for CRISPR are now up to 50%. Not surprisingly, PNAs are not yet able to compete.

But PNAs have some advantages: in Ly, Saltzman, and Glaser’s method, they do not cut the genomic DNA target, so compared with CRISPR, the chance of unintended edits may be lower, Saltzman says. And Lu’s PANDA system has a double recognition system, with both the PNA and DNAzyme being sequence-specific, which should also limit off-target effects. And despite the delivery challenges PNAs face, they are still much smaller than the Cas9 enzyme. That may mean PNAs can access genes in tightly packed regions of the genome that CRISPR enzymes can’t quite squeeze into.

As these applications become more concrete, some hopefuls think the long wait for PNA therapeutic applications may soon be over. “The field is certainly advancing,” Appella says. “These molecules are so unique compared to what’s in nature and wrestling with that is both interesting and challenging.”

*Rachel Brazil is a freelance contributor to**Chemical & Engineering News**, an independent news outlet of the American Chemical Society.*

